# Dr. Samuel B. Baker

**Published:** 1913-07

**Authors:** 


Dr. Samuel B. Baker of Rochester died Monday, May 26, 1913, of heart disease. He leaves a wife and two children. He was but 44 years of age. Born in Lima, N. Y., graduated in University of Buffalo, 1889, and thereupon settled in Rochester. He was a man of very genial, happy, retiring disposition, well liked, and having a very large practice.

